# Reprogramming Atherosclerosis: Precision Drug Delivery, Nanomedicine, and Immune-Targeted Therapies for Cardiovascular Risk Reduction

**DOI:** 10.3390/pharmaceutics17081028

**Published:** 2025-08-07

**Authors:** Paschalis Karakasis, Panagiotis Theofilis, Panayotis K. Vlachakis, Konstantinos Grigoriou, Dimitrios Patoulias, Antonios P. Antoniadis, Nikolaos Fragakis

**Affiliations:** 1 Second Department of Cardiology, Hippokration General Hospital, Medical School, Aristotle University of Thessaloniki, Konstantinoupoleos 49, 54642 Thessaloniki, Greece; aantoniadis@gmail.com (A.P.A.); fragakis.nikos@googlemail.com (N.F.); 2 First Cardiology Department, School of Medicine, Hippokration General Hospital, National and Kapodistrian University of Athens, 11527 Athens, Greece; panos.theofilis@hotmail.com (P.T.); vlachakispanag@gmail.com (P.K.V.); 3 Department of Pharmacology, University of Athens, 75 Mikras Asias Avenue, 11527 Goudi, Greece; dinosgrigoriou@gmail.com; 4 Second Propedeutic Department of Internal Medicine, Faculty of Medicine, School of Health Sciences Aristotle, University of Thessaloniki, 54124 Thesaloniki, Greece; dipatoulias@gmail.com

**Keywords:** atherosclerosis, targeted drug delivery, nanoparticles, atheromatous plaque, endothelial dysfunction, macrophage reprogramming, theranostics, nanomedicine, precision medicine

## Abstract

Atherosclerosis is a progressive, multifactorial disease driven by the interplay of lipid dysregulation, chronic inflammation, oxidative stress, and maladaptive vascular remodeling. Despite advances in systemic lipid-lowering and anti-inflammatory therapies, residual cardiovascular risk persists, highlighting the need for more precise interventions. Targeted drug delivery represents a transformative strategy, offering the potential to modulate key pathogenic processes within atherosclerotic plaques while minimizing systemic exposure and off-target effects. Recent innovations span a diverse array of platforms, including nanoparticles, liposomes, exosomes, polymeric carriers, and metal–organic frameworks (MOFs), engineered to engage distinct pathological features such as inflamed endothelium, dysfunctional macrophages, oxidative microenvironments, and aberrant lipid metabolism. Ligand-based, biomimetic, and stimuli-responsive delivery systems further enhance spatial and temporal precision. In parallel, advances in in-silico modeling and imaging-guided approaches are accelerating the rational design of multifunctional nanotherapeutics with theranostic capabilities. Beyond targeting lipids and inflammation, emerging strategies seek to modulate immune checkpoints, restore endothelial homeostasis, and reprogram plaque-resident macrophages. This review provides an integrated overview of the mechanistic underpinnings of atherogenesis and highlights state-of-the-art targeted delivery systems under preclinical and clinical investigation. By synthesizing recent advances, we aim to elucidate how precision-guided drug delivery is reshaping the therapeutic landscape of atherosclerosis and to chart future directions toward clinical translation and personalized vascular medicine.

## 1. Introduction

Atherosclerosis is a progressive, multifactorial disease characterized by lipid accumulation, chronic inflammation, endothelial dysfunction, and maladaptive vascular remodeling, ultimately leading to plaque formation and clinical events such as myocardial infarction and stroke [1,2,3,4]. Over recent years, the understanding of atherosclerosis has evolved from a focus on systemic lipid dysregulation to a sophisticated view of the disease as a complex interaction between immune, metabolic, and biomechanical pathways operating within a dynamic vascular microenvironment [2,5,6,7].

This paradigm shift has spurred the development of targeted drug delivery strategies designed to modulate atherosclerotic plaques at the molecular and cellular level [8,9,10,11,12,13]. By enabling the localized delivery of therapeutic agents, ranging from small molecules and biologics to nucleic acids, targeted systems seek to enhance treatment efficacy while minimizing systemic exposure and off-target effects. A wide range of nanocarrier systems, including lipid-based vectors (e.g., liposomes and exosomes), polymeric nanoparticles, and hybrid constructs such as metal–organic frameworks (MOFs), have been developed to selectively target key pathological components of atherosclerotic plaques [14,15,16,17]. These platforms vary in terms of size, surface properties, cargo capacity, and release profiles, enabling tailored delivery to inflamed endothelium, dysfunctional macrophages, oxidative stress zones, and lipid-laden regions. Their complementary mechanisms of action offer a versatile toolkit for addressing the multifactorial nature of atherosclerosis and form the foundation of precision-guided therapeutic approaches [14,15,16,17]. These strategies are complemented by advances in ligand-based targeting, biomimetic delivery, and stimuli-responsive systems capable of releasing cargo in response to specific microenvironmental cues. In parallel, innovations in in-silico modeling, imaging-guided delivery, and multifunctional theranostic platforms are expanding the therapeutic possibilities of targeted drug delivery in atherosclerosis [18,19]. Collectively, these approaches are redefining the potential to intervene in disease progression and plaque destabilization with unprecedented precision.

This review aims to provide an integrated overview of emerging targeted drug delivery strategies for atherosclerosis, with a focus on delivery platforms and targeting mechanisms designed to modulate key pathological features such as endothelial inflammation, lipid accumulation, oxidative stress, immune cell infiltration, and extracellular matrix remodeling.

## 2. Mechanistic Pathways Driving Atherogenesis

Atherosclerosis represents a chronic, dynamic, and highly orchestrated pathological process that arises from the interplay between systemic risk factors and maladaptive vascular responses [20,21]. Central to its initiation is the dysfunction of endothelial cells that normally maintain vascular homeostasis and barrier integrity [22,23]. A broad array of injurious stimuli, including hypertension, dyslipidemia, smoking, and hyperglycemia, disrupt endothelial function, rendering the arterial intima permeable to circulating lipoproteins and priming it for inflammatory activation [24]. A central event in early atherogenesis is the subendothelial accumulation of oxidized low-density lipoprotein (oxLDL), which acts as a potent trigger of endothelial activation and a pro-inflammatory milieu [25]. Activated endothelial cells secrete adhesion molecules, cytokines, and chemokines that orchestrate the recruitment of circulating monocytes to sites of vascular injury [26]. Upon transmigration into the intima, monocytes differentiate into macrophages that internalize oxLDL via scavenger receptors, forming foam cells, a hallmark of nascent atherosclerotic lesions.

Foam cells not only perpetuate local inflammation through the release of additional cytokines and matrix-degrading enzymes, but also contribute to ongoing lipid accumulation, thereby amplifying plaque burden. Concomitantly, vascular smooth muscle cells (SMCs) migrate from the media into the intima, undergoing phenotypic modulation characterized by increased extracellular matrix (ECM) synthesis [27]. The resultant deposition of matrix proteins, such as collagen, contributes to the formation of a fibrous cap that transiently stabilizes the growing plaque [27,28]. However, persistent inflammatory signaling, oxidative stress, and protease activity progressively weaken this cap, rendering the plaque increasingly prone to rupture.

Plaque rupture exposes thrombogenic material to circulating blood components, thereby precipitating platelet activation, thrombus formation, and potentially life-threatening ischemic events such as myocardial infarction and stroke [29]. Importantly, this pathogenic continuum of atherogenesis unfolds through three interconnected stages: (i) plaque initiation, marked by endothelial injury and lipid accumulation [30]; (ii) plaque progression, characterized by foam cell formation, SMC migration, and ECM remodeling [31]; and (iii) plaque rupture, culminating in thrombosis and acute vascular events [32].

### 2.1. Inflammatory Mechanisms in Atherogenesis

Atherosclerosis is now firmly established as a chronic, immune-driven inflammatory disorder of the arterial wall [33,34,35,36]. Endothelial dysfunction, induced by traditional risk factors such as hyperlipidemia, hypertension, diabetes, and smoking, serves as the initiating trigger by increasing endothelial permeability and promoting subintimal retention of low-density lipoproteins (LDL) [37,38,39]. In addition to these classical risk factors, mounting evidence suggests that chronic infections contribute to endothelial dysfunction and atherogenesis through both direct and indirect mechanisms [40]. Pathogens such as cytomegalovirus (CMV) [41,42], Chlamydia pneumoniae [43,44], Helicobacter pylori [45], and periodontal bacteria [46] have been associated with increased cardiovascular risk and vascular inflammation. These organisms may promote endothelial activation via multiple pathways, including direct invasion of endothelial cells, induction of pro-inflammatory cytokines (e.g., IL-6 and TNF-α), generation of reactive oxygen species, and upregulation of adhesion molecules. Moreover, molecular mimicry and immune cross-reactivity can perpetuate vascular injury and leukocyte recruitment. Persistent infections may therefore act as chronic inflammatory stimuli, exacerbating endothelial dysfunction and accelerating the initiation of atherosclerotic lesions, particularly in synergy with metabolic and hemodynamic stressors [47]. Once sequestered in the intima, LDL particles undergo oxidative modification, generating oxLDL, which exerts potent immunostimulatory effects. OxLDL activates both endothelial and vascular smooth muscle cells, inducing the expression of adhesion molecules (VCAM-1, ICAM-1), chemokines (MCP-1), and pro-inflammatory cytokines (IL-1β, TNF-α) [48,49,50]. This cascade facilitates the recruitment of circulating monocytes, which differentiate into macrophages upon intimal infiltration.

Macrophages internalize oxLDL through scavenger receptors (CD36 and SR-A), forming foam cells, the histopathologic hallmark of the developing atherosclerotic lesion. Foam cells perpetuate local inflammation through the secretion of pro-inflammatory cytokines (IL-6, IL-1β), reactive oxygen species, and matrix metalloproteinases, thereby enhancing lipid deposition and extracellular matrix remodeling [51,52,53,54,55]. Concomitantly, smooth muscle cell migration and phenotypic modulation further contribute to plaque growth and complexity. The adaptive immune response exerts a critical amplifying role in plaque inflammation. CD4+ T lymphocytes, particularly Th1 and Th17 subsets, are activated by antigen-presenting cells displaying oxLDL-derived and other modified self-antigens within the plaque milieu [52,53]. Th1-derived interferon-γ and Th17-derived IL-17 promote macrophage activation and endothelial dysfunction, while B cells contribute through both antibody production and cytokine release. The resulting immune cell crosstalk sustains a chronic inflammatory state within the plaque microenvironment.

Progression toward clinically dangerous lesions is characterized by a failure of resolution pathways and destabilization of the fibrous cap. Persistent inflammation promotes matrix degradation, apoptosis of SMCs, and necrotic core expansion. When the integrity of the fibrous cap is compromised, exposure of highly thrombogenic material initiates platelet activation and thrombus formation, precipitating acute cardiovascular events such as myocardial infarction or stroke [56,57,58].

Given the central role of inflammation in all stages of atherogenesis, targeting immune pathways represents a promising avenue for therapeutic intervention [59,60,61,62]. Pioneering trials, such as CANTOS (targeting IL-1β) and COLCOT (targeting broader inflammasome activation with colchicine), have provided proof-of-concept that selective anti-inflammatory therapy can reduce cardiovascular event rates independent of lipid lowering [63,64,65]. In parallel, advanced nanomedicine strategies offer novel opportunities for highly targeted immunomodulation. Nanocarriers engineered to engage macrophage-specific receptors (CCR2, CD36, mannose receptor) can deliver anti-inflammatory agents, gene silencing constructs, or polarization modulators to selectively attenuate M1 macrophage responses and promote M2 reparative phenotypes [63]. Moreover, multifunctional nanocarriers capable of co-delivering anti-inflammatory agents, antioxidants, and lipid-lowering drugs may provide synergistic stabilization of vulnerable plaques.

Future research priorities include elucidating the dynamic interplay between innate and adaptive immune mechanisms across different phases of atherogenesis, developing sensitive biomarkers to guide immunomodulatory therapy, and refining the delivery of cell-specific nanotherapies to achieve precision modulation of the vascular immune microenvironment [66,67,68].

### 2.2. Oxidative Stress as a Central Driver of Atherogenesis

Oxidative stress has emerged as a central pathophysiological driver of atherosclerosis, contributing to both the initiation of early lesions and the progression toward clinically significant plaque instability [69,70]. It arises when endogenous antioxidant defense systems are unable to neutralize the excessive generation of reactive oxygen species (ROS), resulting in a net pro-oxidant state that promotes cellular and molecular injury within the vascular wall [71]. One critical consequence of this imbalance is the oxidative modification of LDL, yielding oxLDL, a key pathogenic trigger in early atherogenesis. OxLDL is avidly recognized by scavenger receptors expressed on macrophages, driving their transformation into foam cells and perpetuating lipid accumulation within the arterial intima [72].

Beyond its role in lipid oxidation, oxidative stress serves as a potent amplifier of vascular inflammation through the activation of redox-sensitive transcription factors. Notably, nuclear factor-κB (NF-κB) and activator protein-1 (AP-1) are activated in response to ROS, orchestrating the transcription of numerous pro-inflammatory cytokines, chemokines, and adhesion molecules that further exacerbate plaque progression [73]. Sustained oxidative stress also promotes endothelial dysfunction, augments vascular smooth muscle cell (SMC) migration and proliferation, and drives maladaptive extracellular matrix remodeling, all of which contribute to arterial wall thickening and the evolution of vulnerable plaques [74]. Mitochondrial dysfunction is intricately linked to the pathogenesis of oxidative stress in atherosclerosis, given that mitochondria represent a major source of intracellular ROS. Mitochondrial impairment not only amplifies ROS production but also propagates a feed-forward loop of oxidative damage, inflammation, and cell death, thereby accelerating atherogenic processes [75].

A substantial body of experimental and clinical evidence has established oxidative stress as a central mediator of vascular inflammation and atherogenesis [76,77,78]. Endothelial cells, macrophages, and vascular smooth muscle cells generate reactive oxygen species (ROS) through enzymatic sources such as NADPH oxidases, uncoupled endothelial nitric oxide synthase (eNOS), and mitochondrial dysfunction [79,80,81]. Excessive ROS not only oxidize lipoproteins—facilitating the formation of oxLDL—but also impair nitric oxide bioavailability, promote endothelial activation, and amplify the expression of adhesion molecules and pro-inflammatory cytokines [82,83,84,85]. Moreover, redox-sensitive transcription factors such as NF-κB and AP-1 play key roles in orchestrating inflammatory gene expression in response to oxidative cues [86,87]. Chronic oxidative stress also contributes to foam cell formation, matrix degradation, and necrotic core expansion, thereby accelerating lesion progression and destabilization [88,89]. Collectively, these findings underscore the pathogenic synergy between oxidative stress and inflammation in atherosclerosis and provide a rationale for redox-targeted therapeutic strategies.

Therapeutic strategies aimed at mitigating oxidative stress, such as the use of antioxidants or inhibitors of ROS-generating enzymes, have demonstrated considerable promise in preclinical models [90]. Such interventions have been shown to attenuate lesion formation, reduce plaque instability, and improve endothelial function. However, clinical translation has thus far been disappointing, with large trials of systemic antioxidant therapy yielding limited efficacy. These outcomes underscore the complexity of oxidative stress pathways in vivo and highlight the need for more refined, targeted therapeutic approaches [91].

Nanomedicine offers a compelling platform to overcome many of these translational barriers. Functionalized nanocarriers can be engineered to selectively deliver antioxidant agents (e.g., vitamin E and resveratrol) to sites of vascular inflammation, enabling localized ROS neutralization while minimizing systemic off-target effects [70]. In addition, multifunctional nanocarriers can co-deliver anti-inflammatory agents, matrix metalloproteinase inhibitors, and endothelial-protective compounds to address multiple facets of oxidative plaque progression [70]. Targeting strategies that exploit macrophage-specific markers, activated endothelial cell receptors, or components of the destabilized plaque microenvironment can further enhance the precision and therapeutic efficacy of these nanocarriers [70]. Nonetheless, significant challenges remain, particularly with respect to optimizing tissue penetration, circumventing immune clearance, and achieving controlled drug release within complex vascular lesions [70].

Future research must focus on elucidating the interplay between oxidative stress and other key pathological processes in atherosclerosis, including inflammation, mitochondrial dysfunction, and immune activation, in order to guide the rational design of next-generation, nanomedicine-based interventions. Integrating mechanistic insights with advanced drug delivery technologies holds considerable potential to transform the therapeutic landscape for atherosclerotic cardiovascular disease.

### 2.3. Dysregulated Lipid Metabolism in Atherogenesis

Aberrations in lipid metabolism constitute a fundamental pathogenic axis in the initiation and progression of atherosclerosis [92,93,94]. Under physiological conditions, lipid homeostasis is tightly regulated through dynamic interactions between lipoprotein synthesis, transport, and clearance pathways, ensuring a critical balance between pro-atherogenic LDL-C and protective HDL-C levels [95,96]. Disruption of this equilibrium—characterized by elevated circulating LDL-C and/or diminished HDL-C—facilitates excessive lipid accumulation within the arterial intima, thereby promoting atherogenesis.

Elevated LDL-C levels promote transendothelial transport of LDL particles into the subendothelial space, where they undergo oxidative, glycation, and enzymatic modifications [97,98,99]. Oxidized LDL and other modified lipids serve as potent inducers of endothelial activation, smooth muscle cell (SMC) dysfunction, and macrophage-driven foam cell formation—key cellular events that drive plaque formation and progression. Simultaneously, reductions in HDL-C impair reverse cholesterol transport (RCT), a critical mechanism for the mobilization and clearance of cholesterol from peripheral tissues and plaques [100,101,102,103]. Impaired HDL functionality further exacerbates vascular inflammation and oxidative stress, increasing susceptibility to plaque destabilization and rupture. Importantly, dysregulated lipid metabolism not only initiates early lesion formation but also contributes to plaque vulnerability and the occurrence of acute cardiovascular events. Accumulation of necrotic lipid cores, coupled with defective efferocytosis and persistent inflammation, fosters a milieu conducive to fibrous cap thinning, matrix degradation, and eventual plaque rupture, key drivers of myocardial infarction and stroke [104].

Advances in targeted drug delivery offer promising opportunities to modulate lipid metabolism with enhanced precision and therapeutic efficacy [93]. The rational design of nanomedicine platforms, including nanoparticles, liposomes, and antibody-conjugated carriers, enables site-specific delivery of lipid-lowering agents to atherosclerotic plaques, thereby minimizing systemic toxicity [93]. Strategies targeting LDL receptors, apolipoprotein B (ApoB), or inflamed vascular regions can selectively reduce LDL-C burden and attenuate associated inflammatory responses [93]. Moreover, integrating genomics and biomarker-guided approaches may facilitate the personalization of lipid-lowering therapies, optimizing therapeutic responses in individual patients [93]. Combinatorial drug delivery systems capable of co-delivering lipid-lowering agents, antioxidants, and anti-inflammatory drugs represent a rational strategy to simultaneously address multiple pathological processes within the atherogenic milieu [93].

Looking ahead, the development of next-generation “smart” drug delivery systems holds considerable promise. Such systems could enable dynamic, feedback-responsive modulation of drug release based on real-time assessment of local plaque biology or systemic biomarkers, thereby offering a more adaptive and precise therapeutic paradigm for patients with atherosclerotic cardiovascular disease.

### 2.4. Mitochondrial Dysfunction in Atherogenesis

Mitochondrial dysfunction has emerged as a critical and underappreciated driver of atherosclerosis pathogenesis [105]. Mitochondria serve as the central hubs of cellular energy metabolism, integrating oxidative phosphorylation, redox homeostasis, and apoptotic signaling. Disruption of mitochondrial function profoundly alters cellular metabolism and promotes a pro-inflammatory vascular environment, contributing to both plaque initiation and progression [106]. Several interrelated mechanisms contribute to mitochondrial dysfunction in the atherosclerotic vessel wall [107]. Persistent oxidative stress can damage mitochondrial DNA (mtDNA), leading to impaired transcription of electron transport chain components and inefficient oxidative phosphorylation [108]. Inflammatory cytokines and lipid mediators disrupt mitochondrial membrane potential and promote the opening of the mitochondrial permeability transition pore, resulting in calcium overload and enhanced ROS generation [109]. Additionally, defective mitophagy impairs the clearance of dysfunctional mitochondria, further amplifying oxidative injury [109,110,111,112,113]. These processes create a self-reinforcing cycle in which mitochondrial impairment exacerbates redox imbalance, inflammation, and apoptotic signaling, thereby accelerating vascular injury and plaque progression [112]. During atherogenesis, multiple vascular and immune cell populations, including endothelial cells, vascular smooth muscle cells (SMCs), and macrophages, exhibit varying degrees of mitochondrial dysfunction. Impaired mitochondrial oxidative phosphorylation leads to excess production of ROS, which in turn exacerbates oxidative stress and promotes the oxidative modification of LDL into its pro-atherogenic oxLDL [114,115,116]. Elevated ROS also directly impairs endothelial integrity, promotes endothelial activation, and facilitates monocyte adhesion and transmigration, thereby amplifying early inflammatory responses in the arterial wall.

Mitochondrial dysfunction also exerts profound effects on cell fate decisions within the plaque. Dysregulated mitochondrial signaling promotes apoptosis of endothelial cells, SMCs, and macrophages [117]. The accumulation of apoptotic and necrotic debris contributes to the formation of a necrotic core, a key morphological hallmark of advanced, rupture-prone plaques [118]. Moreover, mitochondrial DNA (mtDNA) released from damaged mitochondria can act as a potent damage-associated molecular pattern (DAMP), further stimulating innate immune pathways and sustaining chronic vascular inflammation [119].

Collectively, these processes—ROS-driven oxidative injury, apoptosis-mediated plaque destabilization, and mtDNA-triggered immune activation—position mitochondrial dysfunction as a central mechanistic node in the pathogenesis of atherosclerosis [120,121,122,123]. Accordingly, therapeutic strategies targeting mitochondrial dysfunction represent a promising avenue for intervention.

Among emerging approaches, the development of mitochondria-targeted antioxidants holds particular promise. Compounds such as MitoQ and MitoTEMPO exploit mitochondrial-targeting moieties (e.g., triphenylphosphonium cations) to selectively accumulate within mitochondria, where they neutralize ROS and mitigate oxidative damage [124]. Preclinical studies have demonstrated that these agents can improve endothelial function, reduce oxidative stress, and attenuate atherosclerotic lesion development [125]. Additionally, targeting mitochondrial quality control pathways, including mitophagy and mitochondrial biogenesis, offers further therapeutic potential to restore mitochondrial homeostasis and improve vascular health [125].

Future research should prioritize delineating the cell-type–specific contributions of mitochondrial dysfunction across distinct stages of atherogenesis, exploring the interplay between mitochondrial signaling and innate immunity, and refining mitochondria-targeted therapeutics for clinical translation. As our understanding of mitochondrial biology in vascular disease deepens, precision targeting of mitochondrial dysfunction may emerge as a transformative strategy to reduce the burden of atherosclerotic cardiovascular disease.

### 2.5. Endothelial Dysfunction in Atherogenesis

Endothelial dysfunction plays a central role in the initiation and progression of atherosclerosis and represents a highly promising target for precision drug delivery [126,127,128,129,130]. The vascular endothelium maintains vascular homeostasis by regulating vasomotor tone, permeability, leukocyte adhesion, and thrombosis, largely through the balanced production of vasodilators such as nitric oxide (NO) and the maintenance of an anti-inflammatory surface [126]. However, exposure to cardiovascular risk factors, including elevated blood pressure, dyslipidemia, cigarette smoking, and hyperglycemia, induces endothelial dysfunction, which is characterized by reduced NO bioavailability, increased oxidative stress, and a shift toward a pro-inflammatory and pro-thrombotic phenotype [126].

Endothelial dysfunction increases vascular permeability, facilitating the subendothelial deposition of LDL and promoting its oxidative modification to oxLDL, a key trigger of atherogenesis [131]. Concurrently, endothelial cells upregulate adhesion molecules such as E-selectin, VCAM-1, and ICAM-1, which promote the recruitment of monocytes and other immune cells into the intima [131,132]. These processes initiate a self-perpetuating cycle of lipid accumulation, inflammation, and plaque development [131]. The loss of endothelial elasticity and vasodilatory capacity further exacerbates vascular dysfunction and accelerates disease progression [131,133].

Given its pivotal role in early atherogenesis, endothelial dysfunction offers an attractive target for nanomedicine-based therapeutic interventions. Nanoparticles functionalized with targeting ligands, such as antibodies or peptides recognizing endothelial activation markers (e.g., E-selectin and ICAM-1) or integrin-binding peptides (e.g., RGD sequences), can selectively accumulate at sites of endothelial injury [134]. This strategy enables localized delivery of therapeutic payloads, including antioxidants, NO donors, anti-inflammatory agents, or gene therapies, thereby restoring endothelial function and attenuating downstream atherogenic cascades [134].

Moreover, nanocarriers can be designed to achieve dynamic, stimuli-responsive drug release in response to local oxidative stress or inflammatory signals within the diseased endothelium [135]. Such precision targeting may enhance therapeutic efficacy while minimizing off-target effects on healthy tissues [135]. For example, recent studies have demonstrated that nanoparticles delivering NO donors or mitochondrial-targeted antioxidants can improve endothelial function, reduce leukocyte adhesion, and suppress plaque progression in preclinical models [135].

Future advancements should focus on optimizing the specificity and stability of endothelial-targeted nanocarriers, exploring novel targeting ligands and delivery platforms, and integrating biomarker-driven strategies to guide patient selection and monitor therapeutic response. Targeted restoration of endothelial homeostasis through nanomedicine offers a promising paradigm for the prevention and treatment of atherosclerotic cardiovascular disease.

### 2.6. Role of Hemodynamic Forces in Atherosclerotic Plaque Development and Destabilization

A growing body of evidence implicates local hemodynamic forces—particularly disturbed flow and low wall shear stress (WSS)—as central determinants in the spatial heterogeneity of atherosclerotic plaque formation and evolution [136,137,138]. The patchy distribution of atherosclerotic lesions, classically observed at arterial bifurcations, curvatures, and branch points, cannot be fully explained by systemic risk factors alone [139]. Instead, these regions are characterized by complex flow patterns, including flow separation and oscillatory shear, which exert pathologically low or fluctuating WSS on the endothelial surface [136]. Caro et al. [140] were among the first to establish the “low shear stress theory” of atherogenesis, demonstrating that early atherosclerotic lesions preferentially develop in arterial regions subjected to low tangential forces, where impaired mass transport, lipid accumulation, and endothelial dysfunction coalesce to form a pro-atherogenic microenvironment.

More recently, Yang and colleagues highlighted that disturbed flow not only modulates endothelial cell alignment and nitric oxide bioavailability but also activates a cascade of inflammatory signaling pathways, including NF-κB and MAPK, that promote leukocyte adhesion, oxidative stress, and prothrombotic states [141]. These hemodynamic influences extend beyond lesion initiation, affecting plaque morphology and stability. Regions of low and oscillatory WSS are enriched in lipid-laden, thin-cap fibroatheromas, whereas abrupt transitions to high shear may exacerbate plaque erosion or rupture [141]. This mechanobiological framework has significant implications for targeted drug delivery: nanoparticles engineered to sense or respond to shear conditions could enable preferential accumulation in flow-disturbed zones, enhancing site-specific therapeutic efficacy. Therefore, incorporation of hemodynamic profiling into nanomedicine design may offer a precision approach to plaque stabilization and vascular risk reduction.

### 2.7. Mechanisms of Atherosclerotic Plaque Progression and Opportunities for Targeted Therapeutic Intervention

Atherosclerotic plaque formation is a dynamic, multistep process that evolves over time through the interplay of lipid accumulation, chronic inflammation, and maladaptive tissue remodeling [142]. The earliest detectable lesions—fatty streaks—arise when endothelial dysfunction permits the subendothelial infiltration of LDL particles. Oxidative and enzymatic modification of LDL leads to the generation of oxLDL, which drives local inflammatory responses and promotes the recruitment of monocytes into the intima. These monocytes differentiate into macrophages, internalize oxLDL, and transform into foam cells, establishing the initial lipid-rich lesion [143].

As the disease progresses, sustained lipid deposition and persistent immune activation amplify the inflammatory milieu within the plaque. Activated macrophages and other immune cells secrete cytokines, chemokines, and matrix-degrading enzymes, which perpetuate cellular dysfunction and matrix remodeling [144]. Concurrently, vascular smooth muscle cells (SMCs) migrate into the intima, where they contribute to fibrous cap formation and extracellular matrix synthesis [144]. Over time, plaques undergo further remodeling, including calcification, fibrous cap thinning, necrotic core expansion, and progressive luminal narrowing [144].

A particularly dangerous aspect of advanced plaque progression is the development of vulnerable plaques characterized by a thin fibrous cap, large necrotic core, and intense inflammatory activity. Such plaques are prone to rupture, exposing thrombogenic material to circulating blood. This precipitates acute thrombus formation, which can rapidly occlude the arterial lumen and trigger life-threatening cardiovascular events such as myocardial infarction and stroke [145,146]. Thus, atherosclerotic plaques represent not only a structural obstruction but also a dynamic and unstable substrate for acute vascular syndromes.

Given the central role of inflammation and immune dysregulation in plaque progression and destabilization, nanomedicine offers compelling opportunities for precision-targeted therapeutic intervention. Nanocarriers can be engineered to deliver anti-inflammatory agents, such as inhibitors of interleukin-1β (IL-1β), tumor necrosis factor-α (TNF-α), and interleukin-6 (IL-6), directly to inflamed plaques [63]. For example, nanoparticles can encapsulate monoclonal antibodies or small-molecule inhibitors to disrupt cytokine-receptor interactions, thereby promoting plaque stabilization while minimizing systemic immunosuppression [63].

In addition, macrophages exhibit intrinsic phagocytic activity and can efficiently internalize nanocarriers [147,148,149]. This property can be exploited to selectively deliver therapeutic payloads, including statins, anti-inflammatory agents, or microRNAs (miRNAs) that modulate macrophage phenotype and function, directly to the plaque microenvironment [148]. Such targeted strategies can attenuate macrophage-driven inflammation, enhance efferocytosis, and promote the resolution of inflammation, ultimately contributing to plaque stabilization [147,148,149].

In sum, atherosclerotic plaque progression is governed by a complex interplay of lipid accumulation, chronic inflammation, matrix remodeling, and calcification [150]. Integrating lifestyle interventions with advanced nanomedicine-based therapies holds considerable promise for combating the burden of atherosclerotic cardiovascular disease. Ongoing research should focus on optimizing targeted delivery platforms, identifying novel molecular targets within vulnerable plaques, and translating these approaches into clinically effective therapies.

## 3. Emerging Therapeutic Strategies and Targeted Drug Delivery in Atherosclerosis

Although substantial progress has been made in understanding and treating atherosclerosis, cardiovascular disease remains the leading cause of global mortality [26,151,152,153,154]. Accordingly, new therapeutic strategies and molecular targets continue to be explored through both preclinical research and large-scale clinical trials. Among these, targeted drug delivery systems are gaining considerable attention for their potential to enhance therapeutic precision and efficacy [152].

Targeted drug delivery strategies in atherosclerosis are designed to enhance therapeutic precision by directing pharmacologic agents specifically to diseased vascular regions. These approaches aim to localize anti-inflammatory compounds, lipid-lowering agents, and pathway-specific inhibitors at sites of plaque development, thereby reducing systemic exposure and off-target effects [155]. Leveraging nanomedicine advances, drug carriers can be functionalized with ligands that recognize cellular and molecular markers within the plaque microenvironment, such as scavenger receptors on macrophages, adhesion molecules on activated endothelium, or enzymes involved in matrix remodeling. This enables selective intervention at different stages of plaque evolution, including initiation, inflammatory amplification, and destabilization, ultimately offering a more effective and safer therapeutic paradigm.

Moreover, targeted delivery platforms offer opportunities for personalized intervention, allowing treatment regimens to be tailored according to patient-specific disease phenotypes and molecular profiles [156]. Despite these advances, critical challenges remain, including optimization of carrier design, targeting specificity, and in vivo stability, that must be addressed to fully realize the clinical potential of these technologies [157].

Nevertheless, targeted drug delivery represents a promising frontier in atherosclerosis therapy, with the potential to shift current paradigms toward more effective, individualized, and disease-modifying interventions.

### 3.1. Nanomedicine in Atherosclerosis: Emerging Therapeutic and Theranostic Strategies

Although initially developed for oncologic applications, nanomedicine has rapidly gained traction as a transformative platform for the diagnosis and treatment of atherosclerosis [158,159]. The design and functionalization of nanoparticles for targeted delivery to atherosclerotic plaques is now a highly active area of research, offering new opportunities to address key limitations of conventional pharmacotherapy [159,160].

**Figure 1:**
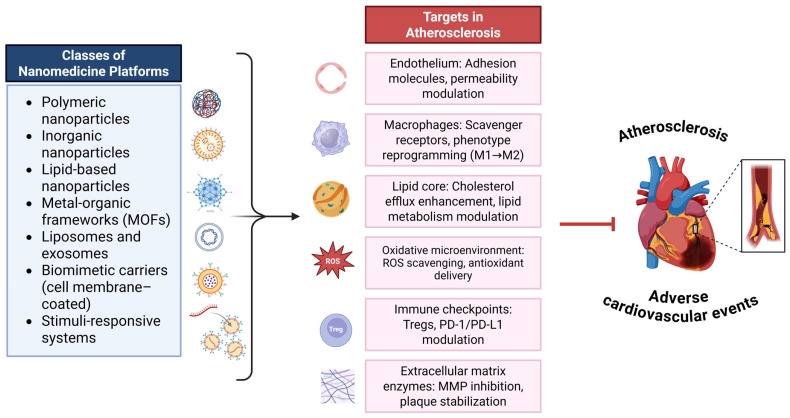
Classes of nanomedicine platforms and their primary biological targets in atherosclerosis. A broad array of advanced delivery systems, including polymeric, inorganic, lipid-based, and MOF nanoparticles; liposomes and exosomes; biomimetic carriers; and stimuli-responsive platforms, are being engineered to engage key pathological features of atherosclerotic plaques. Targeted delivery approaches aim to modulate endothelial dysfunction, reprogram macrophage phenotypes, promote cholesterol efflux from lipid cores, neutralize oxidative microenvironments, regulate immune checkpoint pathways, and inhibit matrix-degrading enzymes involved in plaque destabilization.

Recent studies have demonstrated the capacity of nanomedicine to modulate multiple pathogenic pathways in atherosclerosis. For example, Luo et al. [161] reported that selenopeptide-based nanomedicine attenuates monocyte adhesion and macrophage-mediated inflammation by promoting reactive oxygen species (ROS) clearance and delivering anti-inflammatory agents. This approach achieved a 2.6-fold enhancement in plaque inhibition compared to simvastatin in murine models [161]. Similarly, Li et al. [162] developed a metal-organic cage (MOC)-doped MnO_2_ nanoparticle platform capable of co-delivering hydrogen sulfide (H_2_S) and oxygen (O_2_) to atherosclerotic plaques. This strategy suppressed local inflammation, modulated macrophage polarization, reduced foam cell formation, and improved plaque stability [162]. Figure 1 summarizes key classes of nanomedicine platforms and their principal biological targets in atherosclerosis, highlighting the strategic opportunities for precision modulation of plaque components.

Beyond conventional anti-inflammatory approaches, multifunctional nanoplatforms are being explored to promote plaque regression. Tang et al. [163] introduced the CEZP nanomedicine, which synergistically enhances macrophage efferocytosis, lipid degradation, and cholesterol efflux via the combined effects of zinc ions, epigallocatechin gallate (EGCG), and CpG oligodeoxynucleotides, offering a non-surgical, low-toxicity intervention for advanced plaques [163]. In parallel, Chen et al. (2024) reported that macrophage membrane-coated MnO_2_ nanoparticles (Col@HMnO_2_-MM) selectively target inflamed plaques, scavenge ROS, promote cholesterol efflux, and mitigate foam cell formation, representing a promising strategy for precise, low-toxicity therapy [164].

In addition to these multifunctional systems, specialized platforms such as peptide amphiphile micelles and polyelectrolyte complex micelles are gaining attention for their capacity to address vascular pathology with heightened specificity [165]. Peptide amphiphile micelles have been engineered to target fibrin-rich microenvironments within plaques, particularly concentrating at rupture-prone regions such as the plaque shoulder, enabling co-delivery of imaging agents and therapeutics with spatial precision [165]. Polyelectrolyte complex micelles, formed through electrostatic self-assembly of oppositely charged block copolymers, offer enhanced stability, tunable surface properties, and effective targeting of inflamed endothelium, demonstrating efficacy in attenuating vascular complications in preclinical models [166]. The inclusion of such platforms highlights the evolving landscape of nanotherapeutics tailored to complex atherosclerotic microenvironments.

Nanotheranostics, integrating targeted therapy with diagnostic imaging, are also under active investigation. Such platforms hold potential for identifying vulnerable plaques and guiding personalized interventions. Peng et al. [167] developed osteopontin-modified nanoliposomes (CZALO) encapsulating L-arginine and cerium-zirconium oxide nanoparticles, which promote macrophage reprogramming, modulate nitric oxide bioavailability, exert anti-aging effects on endothelial cells, and enhance vascular homeostasis, all while minimizing systemic toxicity [167].

Despite its promise, several challenges must be addressed before nanomedicine can achieve widespread clinical translation in atherosclerosis. These include improving targeting precision, ensuring long-term biocompatibility and safety, addressing immunogenicity, optimizing manufacturing scalability, and navigating regulatory pathways [168]. Moreover, the stability and pharmacokinetics of nanomedicines in complex vascular environments remain areas of ongoing investigation.

Nevertheless, nanomedicine has emerged as a highly versatile and promising platform capable of addressing the multifactorial nature of atherosclerosis. In contrast to traditional pharmacotherapy, nanoparticle-based delivery systems—encompassing polymeric nanoparticles, liposomes, biomimetic carriers, and nanotheranostic constructs—offer unprecedented opportunities for personalized, site-specific intervention. As reviewed by Hu et al. [169], the field continues to evolve rapidly, with an expanding repertoire of nanomaterials and functionalization strategies aimed at improving therapeutic efficacy and patient outcomes. Table 1 summarizes key nanomaterial-based drug delivery and imaging systems that have demonstrated therapeutic potential in experimental models of atherosclerosis, highlighting their targeting strategies, mechanisms of action, and biological effects on plaque progression and stability.

Ultimately, overcoming existing limitations will be pivotal to unlocking the full clinical potential of nanomedicine in atherosclerosis and advancing the field toward precision, mechanism-guided therapy for this leading cause of global morbidity and mortality.

### 3.2. Immunomodulatory Therapies in Atherosclerosis

#### 3.2.1. Active Immunization Approaches

Active immunization strategies targeting atherosclerosis-related antigens are gaining attention. Vaccination against oxidized LDL and other atherogenic components aims to induce protective immune responses while avoiding autoimmunity [180,181,182,183]. Experimental studies in ApoE^−^/^−^ mice immunized with G3BP2 peptides demonstrated significant reductions in early atherosclerotic plaques, highlighting the potential of antigen-specific immunity [184]. Likewise, peptide-based vaccines targeting oxidized LDL are being explored in clinical trials, though risks of autoantibody generation and autoimmune complications (e.g., systemic lupus erythematosus, rheumatoid arthritis) remain key concerns [185,186].

#### 3.2.2. Regulatory T Cell (Treg)–Enhancing Therapies

Tregs play a pivotal role in maintaining immune tolerance and suppressing vascular inflammation. Experimental models show that enhancing Treg activity mitigates effector Th1-driven responses and prevents plaque progression [60]. Strategies include low-dose IL-2 therapy, CTLA-4 agonism, and efforts to promote IL-37 secretion, all of which foster an anti-inflammatory environment within plaques [187,188,189,190,191]. Clinical trials are underway, though infection risk and immunosuppression must be carefully managed, especially in vulnerable populations (e.g., diabetic or CKD patients). Combination approaches that simultaneously enhance Treg function and restore effector T cell (Teff) homeostasis may offer improved therapeutic balance.

#### 3.2.3. Monoclonal Antibodies and Cytokine Inhibition

Immunotherapies using monoclonal antibodies have already impacted clinical practice. PCSK9 inhibitors (evolocumab, alirocumab) significantly lower LDL-C and reduce atherosclerotic events, although hypersensitivity reactions and injection site issues are noted [180]. IL-1β inhibition with canakinumab demonstrated cardiovascular benefit in the CANTOS trial but also highlighted risks of cytokine release syndrome (CRS), overcompensatory immune activation, and heightened infection susceptibility [186]. CRS remains a key concern for cytokine-targeted therapies, necessitating vigilant patient monitoring.

#### 3.2.4. T Cell–Targeting Therapies

Modulating T cell responses represents a promising yet complex avenue. Targeting immune checkpoints such as PD-1 and CTLA-4 can dampen pathogenic T cell activity within plaques [60]. Anti-PD-1 monoclonal antibodies have been shown to reduce plaque size by suppressing activated PD-1^+^ T cells. Genetic modulation (e.g., CBL-B targeting) influences T cell activation, offering additional therapeutic leverage. Adoptive Treg cell therapies are also advancing, with ex vivo expansion protocols incorporating IL-10 and TGF-β–secreting Tregs [190]. However, challenges remain in achieving precise targeting of T cell subsets while avoiding systemic immunosuppression and autoimmune activation.

#### 3.2.5. B Cell Depletion Strategies

B cells exhibit both pro- and anti-atherogenic functions, depending on subset and context [192,193,194]. B2 cells promote atherosclerosis through pro-inflammatory cytokine production and antigen presentation, while B1 cells may exert protective effects [195,196,197]. Selective B cell depletion with monoclonal antibodies has shown efficacy in reducing plaque size in experimental models of premature vascular aging and diabetes-associated atherosclerosis [192,198,199]. However, long-term consequences of B cell depletion, including impaired humoral immunity, infection risk, and potential malignancy, require careful evaluation [192,193]. Comprehensive immune monitoring will be essential to guide the safe implementation of B cell-targeted therapies.

### 3.3. Anti-Inflammatory Therapies

Over the past two decades, it has become increasingly evident that both innate and adaptive immune responses are integral to the pathogenesis of atherosclerosis. Among clinical biomarkers, C-reactive protein (CRP) is widely used to monitor residual inflammatory risk and guide therapeutic strategies in high-risk patients [200]. Despite promising clinical advances, the limitations and adverse effects associated with anti-inflammatory interventions are only now being fully appreciated.

Inflammasomes, particularly the NLRP3 inflammasome, play a pivotal role in vascular inflammation and plaque progression [201,202,203,204]. A wide range of stimuli, including pathogen-associated molecular patterns (PAMPs), damage-associated molecular patterns (DAMPs), metabolic disturbances, calcified particles, somatic mutations (e.g., TET2 mutations), and environmental insults, can activate inflammasome complexes [205]. Once activated, inflammasomes facilitate the cleavage of pro-caspase-1 into active caspase-1, which in turn processes pro–IL-1β and pro–IL-18 into their mature, bioactive forms. Concurrently, caspase-1 cleaves gasdermin D (GSDMD), whose N-terminal domain forms membrane pores, enabling the release of inflammatory cytokines and promoting pyroptotic cell death.

IL-1β and IL-18 are central mediators of this inflammatory cascade [206]. IL-1β, primarily secreted by activated macrophages, amplifies inflammatory responses by promoting leukocyte recruitment and stimulating further cytokine release [207]. IL-18 complements this activity by driving natural killer (NK) cell and T-cell activation and enhancing interferon-gamma production, which sustains chronic vascular inflammation [208]. While these cytokines are essential for host defense, their overactivation exacerbates plaque inflammation and instability, rendering them attractive therapeutic targets [209].

Pharmacological strategies targeting inflammatory pathways have gained momentum. Although non-steroidal anti-inflammatory drugs (NSAIDs) possess broad anti-inflammatory properties, their clinical utility in atherosclerosis is limited by associations with increased cardiovascular risk [210]. In contrast, cytokine-specific interventions, such as IL-1β inhibition with canakinumab, have demonstrated superior efficacy and safety. The landmark CANTOS trial established that IL-1β blockade not only reduces recurrent cardiovascular events but also lowers systemic inflammation, as reflected by reductions in CRP and other biomarkers [211].

Current research is also focused on directly targeting upstream inflammasome activation, particularly the NLRP3 inflammasome, which orchestrates the release of IL-1β and IL-18 and contributes to plaque destabilization [212]. NLRP3 inhibitors are undergoing clinical evaluation as promising candidates for attenuating vascular inflammation and promoting plaque stabilization [213].

Despite these advances, anti-inflammatory therapy in atherosclerosis remains fraught with challenges. The complexity of inflammatory networks, the heterogeneity of therapeutic targets, risks of immunosuppression, potential drug resistance, economic considerations, and limitations of current clinical trials all warrant careful consideration. Moving forward, a more comprehensive and personalized approach—integrating anti-inflammatory strategies with lipid-lowering therapies, metabolic modulation, and lifestyle interventions—will be essential to effectively address this multifactorial and evolving disease.

### 3.4. mRNA-Based Therapeutics in Atherosclerosis

Messenger RNA (mRNA)–based therapeutics are rapidly emerging as a novel class of interventions with significant potential for the treatment of atherosclerosis and cardiovascular diseases [214]. By enabling transient and controlled expression of therapeutic proteins, mRNA technologies offer a versatile platform to target key molecular pathways involved in vascular inflammation, lipid metabolism, and plaque progression [214].

Recent advances in nanomedicine have enabled the targeted delivery of mRNA constructs to lesional macrophages within atherosclerotic plaques, thereby providing a means to modulate local immune responses and inhibit inflammation. In preclinical models, nanoparticle-mediated delivery of therapeutic mRNA to macrophages has been shown to attenuate plaque inflammation and reduce disease burden [215]. Moreover, cell-specific mRNA strategies are being explored to enhance the precision and efficacy of interventions in cardiovascular diseases, underscoring the potential for highly personalized therapeutic approaches [216].

The use of mRNA therapeutics to modulate both vascular inflammation and lipid metabolism is particularly promising in the context of atherosclerotic cardiovascular disease. For example, mRNA-based delivery of anti-inflammatory cytokines, such as IL-10, via exosome platforms has demonstrated the capacity to suppress plaque inflammation and promote vascular homeostasis [217,218]. Such strategies represent a new paradigm in cardiovascular medicine, complementing existing pharmacotherapies and targeting disease mechanisms that have been difficult to modulate with conventional drugs.

Nevertheless, several critical challenges must be addressed to fully realize the potential of mRNA therapeutics in atherosclerosis. Efficient, cell-specific delivery remains a major hurdle, particularly given the complex architecture of atherosclerotic plaques. In addition, mRNA constructs must be optimized to minimize immunogenicity and ensure stability, while long-term safety profiles remain to be fully elucidated. Regulatory considerations and scalable manufacturing processes will also be essential for clinical translation [219,220,221]. Future directions include the exploration of inflammation-responsive mRNA elements and the role of RNA-binding proteins in regulating the stability and translation of therapeutic mRNAs within diseased vascular tissues [222,223]. Such insights may enable the development of next-generation mRNA therapeutics with enhanced precision, durability, and safety.

## 4. Targeted Therapeutics

### 4.1. Targeted Anti-Inflammatory Strategies

Targeted anti-inflammatory interventions have rapidly emerged as a focal point in the treatment of atherosclerosis, driven by growing recognition of the central role of inflammation in disease initiation, progression, and plaque destabilization [224]. Beyond lipid accumulation, atherosclerosis is now understood as a chronic immune-mediated disorder, in which persistent inflammatory responses orchestrate vascular injury and promote the development of vulnerable plaques [225]. Accordingly, the precise modulation of inflammatory pathways represents a key therapeutic objective aimed at stabilizing plaques and reducing cardiovascular risk.

Current efforts in this field encompass several promising strategies. Targeting pro-inflammatory cytokines—particularly interleukin-1β (IL-1β) and interleukin-18 (IL-18)—has garnered considerable attention [37]. Inhibition of these cytokines mitigates local vascular inflammation and enhances plaque stability. The landmark CANTOS trial demonstrated that IL-1β blockade can significantly reduce major adverse cardiovascular events, thereby validating the therapeutic relevance of this approach.

Parallel research has identified the NLRP3 inflammasome as a critical upstream mediator of cytokine activation and vascular inflammation [226]. Activation of the NLRP3 inflammasome promotes the maturation and release of IL-1β and IL-18, contributing to endothelial dysfunction, macrophage pyroptosis, and plaque progression. Consequently, small-molecule inhibitors targeting NLRP3, such as MCC950, have shown substantial efficacy in preclinical models. MCC950 suppresses inflammasome assembly and activation, thereby attenuating macrophage-driven inflammation and pyroptotic cell death within atherosclerotic lesions [224].

In addition to small molecules, advances in nanomedicine and biologics are facilitating highly precise anti-inflammatory interventions [227,228]. Engineered nanoparticles and targeted biologic carriers enable localized delivery of anti-inflammatory agents directly to inflamed vascular regions, minimizing systemic exposure and enhancing therapeutic efficacy. Such approaches offer the potential to circumvent limitations associated with broad immunosuppression, which remains a key challenge in systemic anti-inflammatory therapy.

### 4.2. Targeted Antioxidant Strategies

Atherosclerosis, the principal underlying cause of cardiovascular disease, is increasingly recognized as a chronic inflammatory condition of the vascular wall, in which oxidative stress plays a pivotal role in driving endothelial dysfunction, lipid oxidation, and plaque instability [229,230]. As such, targeted antioxidant strategies have emerged as an attractive approach to complement conventional therapies and address key pathophysiologic mechanisms of the disease.

Dietary interventions remain a foundational component of cardiovascular prevention [231,232]. Bioactive dietary compounds, such as flavonoids and polyphenols, exert antioxidant and anti-inflammatory effects that may attenuate atherogenesis. Massaro et al. [233] highlighted the impact of nutrient intake on gene expression and signaling pathways implicated in atherosclerotic development. Among these, flavonoids—widely studied in both traditional Chinese medicine and Western nutritional science—have demonstrated vascular protective effects through modulation of macrophage-driven inflammation and oxidative stress [234]. Similarly, antioxidant-rich apple extracts and proanthocyanidin compounds have been shown to mitigate lipid peroxidation products such as 4-hydroxynonenal (4-HNE), thereby preserving vascular function in experimental models [235].

At the molecular level, the Nrf2 pathway has emerged as a central regulator of antioxidant defenses in vascular cells. Activation of Nrf2 and its downstream effector, heme oxygenase-1 (HO-1), induces a cytoprotective transcriptional program that mitigates oxidative damage and inflammation within atherosclerotic plaques. Recent reviews have underscored the therapeutic potential of targeting this pathway to combat atherosclerotic progression [236]. Parallel investigations link lipid peroxidation, oxidative stress, and chronic inflammation in the development of atherosclerosis, highlighting the need for integrated antioxidant and anti-inflammatory interventions [237,238].

Despite promising preclinical data, the clinical translation of antioxidant-based therapies has proven challenging. Systemic antioxidant supplementation has largely failed to demonstrate consistent cardiovascular benefit, owing in part to issues of bioavailability, delivery specificity, and the complexity of redox signaling in vivo [78,239]. As such, the focus is shifting toward targeted antioxidant strategies, leveraging controlled-release formulations, nanocarriers, and biomaterials designed to deliver antioxidant agents selectively to diseased vascular tissues [78,239]. Such approaches aim to overcome limitations of conventional systemic therapy by enhancing local efficacy while minimizing off-target effects.

Emerging work also explores the role of thioredoxin-based systems, anti-aging pathways, and advanced biomaterials in modulating oxidative stress and vascular homeostasis [78,239,240,241]. These innovations hold promise for refining antioxidant strategies and positioning them as effective components of multifaceted therapeutic regimens for atherosclerosis.

In conclusion, while antioxidant modulation of vascular oxidative stress remains a compelling therapeutic target, future success will depend on the development of highly specific, locally active interventions. Advances in delivery technologies, molecular targeting, and systems biology approaches are poised to drive the next generation of antioxidant therapies capable of addressing the complex oxidative landscape of atherosclerotic disease.

### 4.3. Targeted Lipid Control

Aberrant lipid metabolism is a central driver of atherogenesis and represents a critical target for therapeutic intervention [242]. Beyond systemic lipid lowering, recent advances increasingly focus on cell-specific and pathway-specific modulation of lipid handling within the vascular wall and immune cells, aiming to disrupt the vicious cycle of lipid accumulation, inflammation, and plaque progression [20].

Fatty acid-binding proteins (FABPs), particularly FABP4, have emerged as key intracellular regulators of lipid trafficking and inflammatory signaling [243]. FABP4 functions as a lipid chaperone in adipocytes, macrophages, and vascular smooth muscle cells, linking lipid metabolism to the regulation of pro-inflammatory cytokines, insulin resistance, and endothelial dysfunction [243]. Dysregulated FABP4 expression is implicated in obesity, type 2 diabetes mellitus (T2DM), and cardiovascular disease. In the context of atherosclerosis, FABP4 promotes foam cell formation and enhances plaque inflammation, positioning it as a promising therapeutic target [243].

Complementary strategies target lipid accumulation within macrophages and foam cells, which are integral to plaque development. Experimental therapies aimed at modulating foam cell lipid handling and autophagy pathways have shown efficacy in preclinical models [244]. Activation of the Nrf2 pathway with agents such as JC-5411 enhances antioxidant defenses, reduces inflammation, and improves lipid metabolism, collectively attenuating plaque progression in ApoE^−^/^−^ mice [245]. Similarly, dysregulated activity of PCSK6—driven by plaque-associated oxonol and 4-hydroxynonenal (HNE)—has been linked to matrix metalloproteinase-9 (MMP-9) activation and plaque destabilization, highlighting PCSK6 as a potential target for therapeutic intervention [246].

Macrophage autophagy also plays a pivotal role in regulating intracellular lipid homeostasis and resolving inflammation [247]. Disruptions in autophagic flux impair lipid efflux, promote foam cell death, and exacerbate plaque vulnerability. In parallel, the post-transcriptional regulation of lipid metabolism by microRNAs (miRNAs) is gaining increasing attention [247]. The miR family modulates key aspects of cholesterol efflux, lipogenesis, and inflammatory signaling, offering a rich landscape for the development of RNA-based therapeutics [247].

Phytochemicals and natural products provide additional avenues for targeted lipid regulation. Compounds such as QXXZF have been shown to enhance cholesterol efflux and suppress macrophage-mediated inflammation, while rosmarinic acid exhibits lipid-lowering and anti-atherogenic properties through modulation of multiple metabolic pathways [248,249]. Moreover, vitamin D influences lipid metabolism, immune modulation, and endothelial function, collectively contributing to atheroprotection. These pleiotropic effects further underscore the potential of integrated therapeutic strategies targeting lipid regulation and vascular inflammation [250].

### 4.4. Targeting Macrophages

Macrophages are key orchestrators of atherosclerotic lesion development, influencing disease initiation, progression, and plaque stability. Derived from circulating monocytes recruited in response to endothelial injury and lipid accumulation, macrophages are attracted to lesions by chemokines such as monocyte chemoattractant protein-1 (MCP-1) and colony-stimulating factors [251]. Once in the intima, monocytes differentiate into macrophages under the influence of colony-stimulating factor-1 (CSF-1), which sustains their survival and differentiation.

The local plaque microenvironment drives macrophage phenotypic plasticity, giving rise to a spectrum of functional states traditionally classified as pro-inflammatory M1 and anti-inflammatory M2 subsets [252]. M1 macrophages, induced by stimuli such as interferon-gamma (IFN-γ) and lipopolysaccharide (LPS), secrete pro-inflammatory cytokines (TNF-α, IL-1β, IL-6) and reactive oxygen species (ROS), thereby amplifying vascular inflammation and contributing to endothelial dysfunction and plaque instability [253]. Conversely, M2 macrophages, polarized by IL-4 and IL-13, support tissue repair, promote resolution of inflammation, and facilitate efferocytosis—the clearance of apoptotic cells—thus limiting necrotic core formation and enhancing plaque stability [254].

Lipid metabolism is central to macrophage function in atherosclerosis. Macrophages engulf modified lipoproteins—particularly oxLDL—via scavenger receptors (CD36 and SR-A), leading to foam cell formation and early plaque growth [255]. Efficient cholesterol efflux, mediated by transporters such as ABCA1 and ABCG1, is essential to counterbalance lipid uptake. Impaired efflux promotes lipid accumulation, necrotic core development, and plaque vulnerability [256]. Nuclear receptors, notably liver X receptors (LXRs) and peroxisome proliferator-activated receptors (PPARs), regulate genes controlling lipid homeostasis and inflammation. Activation of LXRs enhances cholesterol efflux and suppresses pro-inflammatory gene expression, underscoring their therapeutic potential [257].

Beyond their roles in lipid metabolism, macrophages are central drivers of plaque inflammation and oxidative stress. M1 macrophages generate ROS that oxidize lipoproteins, further promoting foam cell formation and endothelial dysfunction [253]. Simultaneously, macrophage-derived matrix metalloproteinases (MMPs) degrade extracellular matrix components, weakening the fibrous cap and increasing the risk of plaque rupture and thrombotic events [258]. Importantly, defective efferocytosis exacerbates necrotic core formation in advanced lesions, further destabilizing plaques [254].

Conversely, targeting macrophages offers opportunities to enhance plaque stability. M2 macrophages contribute to fibrous cap reinforcement and facilitate cholesterol clearance through efflux pathways [259]. Enhancing M2 polarization and efferocytic capacity represents a promising strategy to counteract plaque progression and promote vascular repair.

In summary, macrophages are pivotal to the immunometabolic regulation of atherosclerosis. The balance between pro-inflammatory and reparative macrophage phenotypes dictates plaque dynamics and clinical outcomes. Therapeutic strategies aimed at modulating macrophage polarization, enhancing cholesterol efflux, and improving efferocytosis hold significant potential to stabilize plaques and prevent acute cardiovascular events. Future research should focus on identifying molecular targets and delivery platforms capable of precisely reprogramming macrophage function within the atherosclerotic niche, thereby offering new avenues for disease modification.

### 4.5. Targeting Enzymes and Modulating the Vascular Microenvironment in Atherosclerosis

Enzyme-based therapies are emerging as a promising frontier in the targeted treatment of atherosclerosis, offering novel opportunities to modulate key pathogenic pathways with high specificity. Recent advances in enzyme-targeted drug delivery platforms have demonstrated the potential to reshape the vascular microenvironment and attenuate disease progression. For instance, Song et al. [260] developed a simvastatin-loaded hyaluronic acid–mesoporous silica nanoparticle (SIM@HA-MSN) system capable of improving the local vascular milieu and enhancing therapeutic efficacy. Similarly, Nguyen et al. [261] identified heparinase as a promising enzymatic target due to its capacity to modulate extracellular matrix remodeling, thereby influencing plaque composition and stability.

The enzymatic regulation of cholesterol biosynthesis is another critical avenue. Mamoudou et al. [262] demonstrated that bioactive peptides derived from enzymatic hydrolysis exhibit inhibitory activity against HMG-CoA reductase, a key enzyme in cholesterol synthesis, highlighting a novel strategy for metabolic modulation in atherosclerosis. Furthermore, reactive oxygen species (ROS)–generating enzymes have been implicated in driving oxidative stress within plaques. Targeting ROS-producing enzymatic pathways has shown therapeutic promise in preclinical models [263]. The combined use of omega-3 fatty acids and coenzyme Q10 has also been reported to exert synergistic anti-atherosclerotic effects in hypercholesterolemic models, further supporting the potential of enzyme-related interventions [264].

Beyond enzymatic targeting, the pathogenesis of atherosclerosis is profoundly influenced by the vascular microenvironment, a dynamic milieu shaped by lipid metabolism, oxidative stress, and mechanical forces [265]. These factors critically modulate macrophage function and contribute to lesion progression. The concept of the perivascular mechanical environment (PVME), introduced by Yamaguchi et al. [266], emphasizes the role of biomechanical forces in shaping atherogenesis. Their findings demonstrated that differential mechanical stress on coronary arteries versus internal thoracic arteries contributes to site-specific susceptibility to atherosclerosis.

In addition to mechanical forces, genetic predisposition and lifestyle factors further shape the internal microenvironment. Variants in apolipoprotein E (ApoE) genotype, coupled with gene-environment interactions, substantially influence individual susceptibility to coronary artery disease [267]. Thus, a comprehensive understanding of the complex interplay between mechanical, genetic, and metabolic factors is essential for developing more effective and personalized therapeutic strategies.

### 4.6. Targeted Modulation of Signaling Pathways

#### 4.6.1. Targeting Co-Stimulatory Pathways

The modulation of co-stimulatory signaling pathways has emerged as a promising immunotherapeutic approach in the treatment of atherosclerosis and related cardiovascular diseases. Co-stimulatory molecules are integral to T-cell activation and immune homeostasis [268]. In the context of atherosclerosis, dysregulation of these pathways contributes to the amplification of maladaptive immune responses, promoting chronic vascular inflammation and driving plaque progression.

Under physiological conditions, T-cell activation requires two signals: antigen presentation via the major histocompatibility complex (MHC) and engagement of co-stimulatory receptors such as CD28 [269]. In atherosclerosis, an imbalance in co-stimulatory and co-inhibitory signaling shifts the immune landscape toward a pro-inflammatory state. Activated T cells secrete cytokines that exacerbate endothelial dysfunction, recruit additional immune cells, and destabilize atherosclerotic plaques [270].

Therapeutic targeting of co-stimulatory pathways offers an opportunity to restore immune balance and attenuate vascular inflammation. Agents such as CTLA-4-Ig (abatacept), which inhibits CD28-mediated co-stimulation, and PD-1 pathway modulators are currently being explored in clinical trials for cardiovascular indications [271]. These interventions not only modulate T-cell activity but have also demonstrated potential in reducing plaque burden and limiting inflammatory responses in preclinical and early clinical studies [271]. Notably, immune checkpoint modulation may provide additive benefits when combined with lipid-lowering and anti-inflammatory therapies.

However, despite the promise of this approach, several challenges remain. Long-term safety, the risk of immunosuppression, and the potential for unintended effects on host defense mechanisms must be rigorously evaluated [272]. Moreover, a deeper understanding of the temporal and spatial dynamics of co-stimulatory signaling in vascular immune responses is needed to inform optimal therapeutic design and patient selection.

#### 4.6.2. Targeting the mTOR Signaling Pathway

The mammalian target of rapamycin (mTOR) signaling pathway is a central regulator of cell metabolism, growth, and survival, and plays a critical role in atherosclerosis [273]. The pathway operates through two distinct complexes: mTORC1 and mTORC2 [274]. mTORC1 controls protein synthesis, lipid metabolism, and autophagy, whereas mTORC2 regulates cell survival and cytoskeletal dynamics. In atherosclerosis, aberrant mTORC1 activation enhances lipogenesis (via SREBP-1c), suppresses autophagy, and promotes lipid and inflammatory accumulation within plaques.

Therapeutic targeting of this pathway has shown promise. Inhibition of mTORC1 reduces plaque area and inflammatory markers in preclinical models [275], while modulation of mTORC2 improves endothelial function and attenuates vascular inflammation [276]. Both complexes are upregulated in advanced plaques [277,278], highlighting their relevance to disease progression.

However, systemic mTOR inhibition can cause immunosuppression and metabolic side effects [279]. Future efforts must focus on optimizing selectivity to maximize efficacy while minimizing adverse outcomes [280]. Overall, targeted modulation of mTOR signaling offers a promising avenue for atherosclerosis therapy [275], but further clinical validation is required.

#### 4.6.3. Targeting the Nrf2/HO-1 Signaling Pathway in Atherosclerosis

The Nrf2/HO-1 signaling axis has emerged as a promising therapeutic target in atherosclerosis due to its potent antioxidant, anti-inflammatory, and cytoprotective effects [281]. Activation of Nrf2 promotes the expression of downstream effectors such as heme oxygenase-1 (HO-1), which mitigates oxidative stress, reduces endothelial injury, and inhibits apoptosis, key mechanisms contributing to plaque stability. Preclinical studies have provided compelling evidence for this pathway’s therapeutic potential. Pharmacologic activation of Nrf2 significantly reduced lesion formation and oxidative damage in atherosclerotic models [282]. Similarly, enhancing Nrf2/HO-1 signaling has been shown to reduce plaque size and improve vascular wall integrity [283].

Despite these promising results, clinical translation faces important challenges. Nrf2 activation exerts broad cytoprotective effects that may inadvertently promote the survival of malignant cells, raising concerns about long-term safety [284]. Thus, the development of tissue-specific Nrf2 activators or targeted delivery systems will be crucial to harness this pathway’s benefits while minimizing potential oncogenic risks.

### 4.7. Other Emerging Therapeutic Strategies

Nanotechnology offers transformative potential for both the diagnosis and treatment of atherosclerosis. Ma et al. [285] reported the development of a reactive oxygen species (ROS)–responsive theranostic nanoplatform incorporating two-photon aggregation-induced emission (AIE) imaging, enabling real-time visualization of plaque oxidative activity alongside dual therapeutic delivery. In parallel, Zheng et al. [286] demonstrated that a biomimetic Sim@PMPB nanoparticle not only stabilized atherosclerotic plaques but also permitted dynamic tracking of H_2_O_2_-mediated oxidative stress within lesions.

Recent advances in in-silico modeling further demonstrate the potential of MOF-based nanocarriers functionalized with P-selectin aptamers for precise drug delivery to atherosclerotic plaques, achieving high surface density and minimizing off-target toxicity under physiologically realistic flow conditions [19]. This highlights the critical role of computationally guided design in optimizing targeted nanomedicine for clinical translation.

Efforts to refine plaque-targeted delivery also extend to the modulation of established pharmacologic targets. While PPARγ agonists exhibit potent anti-inflammatory and metabolic effects, their clinical utility is constrained by systemic side effects [287]. Emerging nanoparticle delivery systems aim to enhance the therapeutic index of such agents. Nasr et al. [288] provided a comprehensive review of nanomedicine innovations designed to optimize plaque targeting and improve clinical outcomes.

Beyond nanotechnology, emerging insights into ferroptosis—a regulated form of iron-dependent cell death—have revealed new therapeutic avenues. Wu et al. [289] identified ferroptosis-related gene signatures in atherosclerosis, highlighting heme oxygenase-1 (HMOX1) as a potential biomarker and therapeutic target [289]. The modulation of ferroptosis pathways may offer novel means to influence plaque composition and stability. Additional innovative approaches include targeting metabolic reprogramming, matrix remodeling, and advanced imaging-guided interventions, as reflected in recent studies [290,291,292,293]. Collectively, these emerging strategies hold promise for enhancing the precision and efficacy of atherosclerosis management. However, further research is needed to validate their clinical applicability and long-term safety.

## 5. Future Directions and Conclusions

Nanomedicine is poised to reshape the therapeutic landscape of atherosclerosis by enabling cell-specific, microenvironment-responsive interventions that directly modulate the pathological processes driving plaque initiation, progression, and rupture. In this review, we have detailed how diverse nanocarrier systems, including liposomes, exosomes, polymeric nanoparticles, and metal–organic frameworks, are being engineered to deliver therapeutic payloads such as anti-inflammatory agents, lipid-modifying drugs, antioxidants, and nucleic acids to sites of vascular injury. These platforms allow for enhanced precision, reduced systemic toxicity, and the potential for real-time imaging through multifunctional theranostic integration. Moreover, ligand-functionalized and biomimetic nanocarriers offer the ability to discriminate between healthy and diseased vascular regions, laying the foundation for a new era of personalized vascular therapy.

Despite this promise, several challenges remain that must be addressed to enable successful clinical translation. First, the regulatory framework for complex nanomedicines is still evolving. Agencies such as the U.S. FDA and EMA require extensive data on long-term safety, reproducibility, and pharmacokinetics, particularly in chronic conditions like atherosclerosis that demand durable, repeatable interventions. This is especially pertinent for agents targeting immune modulation or lipid metabolism, which may carry off-target or immunosuppressive risks in vulnerable populations, including individuals with diabetes, chronic kidney disease, or advanced age [294].

Second, current clinical endpoints often rely on surrogate markers, such as LDL-C reduction or imaging-derived plaque metrics, which, while informative mechanistically, may not sufficiently capture clinical efficacy. As regulatory expectations shift toward hard outcomes (e.g., myocardial infarction, stroke, cardiovascular mortality), future trials must be adequately powered and longitudinally designed to demonstrate real-world therapeutic impact [295,296,297].

Third, the biological heterogeneity of atherosclerosis—modulated by genetic, epigenetic, and environmental factors—necessitates a shift toward precision medicine. Nanomedicine and mRNA-based platforms are well-positioned to support this transition, offering modularity and tunability for patient-specific targeting. However, achieving efficient, cell-specific biodistribution with minimal immunogenicity and optimal pharmacodynamics remains an area of active investigation. Scalable manufacturing processes and cost-effectiveness analyses are also urgently needed to ensure accessibility in diverse healthcare settings.

The atherosclerotic plaque microenvironment itself poses formidable barriers. Effective delivery under dynamic flow, across endothelial barriers, and within complex inflammatory niches requires next-generation biomimetic systems capable of adapting to mechanical and biochemical cues. In-silico modeling and machine learning are expected to play a pivotal role in the rational design and optimization of such platforms. Encouragingly, experimental systems such as P-selectin–functionalized MOFs and shear-sensitive liposomes are already showing promise under physiologic flow conditions.

To accelerate the bench-to-bedside trajectory, future research must prioritize the following pillars: (1) robust preclinical validation in advanced animal models that replicate human plaque biology; (2) engineering of nanocarriers with improved targeting fidelity, biosafety, and pharmacokinetic profiles; (3) design of multi-arm clinical trials powered for major adverse cardiovascular events (MACE); and (4) integration of biomarker-guided, patient-stratified treatment algorithms rooted in the principles of precision medicine.

In conclusion, while atherosclerosis remains a leading global cause of morbidity and mortality, advances in nanomedicine offer a transformative path forward. By coupling mechanistic insight with cutting-edge delivery systems, we are beginning to realize the potential of personalized, site-specific interventions. Unlocking this potential will require a sustained, multidisciplinary effort spanning materials science, vascular biology, computational modeling, and clinical cardiology. If successful, it will not only mitigate the burden of atherosclerotic disease but also redefine therapeutic paradigms in cardiovascular medicine.

## Figures and Tables

**Table 1:** Key Nanomaterials for Anti-Atherosclerosis Therapy: Drug-Carrying Platforms, Targeting Strategies, and Mechanistic Actions.

Nanomaterial	Drug/Agent Carried	Targeting/Physiological Activity	Mechanism of Action	Reference
Multi-walled carbon nanotubes (MWCNTs)	Statins (pitavastatin, atorvastatin, fluvastatin, lovastatin)	Inhibition of IL-1β production in macrophages; targeting NLRP3 inflammasome pathway	Inhibits internalization of MWCNTs and cholesterol crystals into macrophages, suppressing NLRP3-mediated IL-1β release	Cui et al., 2021 [170]
Lipid-based nanoparticles (rHDL NPs)	Cholesteryl esters; hydrophobic and hydrophilic drugs	Targeting macrophages and foam cells; promoting cholesterol efflux; anti-inflammatory effects	Reverse cholesterol transport via ABCA1, ABCG1, SR-B1; reduces lipid burden and inflammation in plaques	Cheng et al., 2023 [171]
Polymeric nanoparticles (PLGA, PEG, PAMAM, etc.)	Various drugs (e.g., rapamycin, dexamethasone, siRNA)	Targeting macrophages, endothelial cells, and foam cells; controlled drug release	Sustained drug release; reduces phagocytosis by RES; enables longer circulation time, and enhances plaque targeting	Cheng et al., 2023 [171]
Biomimetic nanomaterials (Macrophage membrane-coated NPs)	Rapamycin, siRNA, or other drugs	Targeting inflamed plaques and foam cells via macrophage membrane antigens	Inhibits lipid uptake by foam cells; sequesters pro-inflammatory cytokines; enhances circulation time; reduces inflammation	Cheng et al., 2023 [171]
Inorganic nanoparticles (Iron oxide NPs, AuNPs, MSNs)	Imaging agents, antioxidants, drugs	Targeting macrophages, inflamed endothelium, and thrombus	Enables multimodal imaging (MRI, CT, PA), ROS scavenging, phototherapy, targeted drug delivery	Cheng et al., 2023 [171]
mPEG-DSPE Calcium Phosphate (CaP) nanoparticles	Dexamethasone acetate (DEX) and Rapamycin (RAPA)	Targeting atherosclerotic plaques; protection of endothelial cells; foam cell apoptosis; plaque regression	DEX protects endothelial cells from oxidative stress; RAPA induces foam cell apoptosis via autophagy; DR-NPs accumulate at plaques, reduce lipid core and necrotic core size, and downregulate adhesion molecules (MMP-2, MMP-9, ICAM-1)	Luo et al., 2020 [172]
Cargo-switching nanoparticles (CSNP) with cyclodextrin–statin core and phospholipid shell	Simvastatin; cyclodextrin as cholesterol scavenger	Targeting cholesterol-rich atherosclerotic plaques; reducing plaque cholesterol and macrophages; promoting plaque regression	Cyclodextrin binds cholesterol in plaque, displacing statin; statin is released locally → anti-inflammatory and antiproliferative effects; cholesterol is scavenged from plaques; synergistic effect	Kim et al., [173]
Lipid-polymer hybrid nanoparticles (PLGA core + lipid-PEG shell + S2P peptide)	siRNA targeting Camk2g (CaMKIIγ)	Targeting macrophages in atherosclerotic plaques; reduction of plaque necrosis; promotion of fibrous cap stability; increased efferocytosis	siRNA silencing of Camk2g in plaque macrophages → restores MerTK-mediated efferocytosis → reduces necrotic core size; increases fibrous cap thickness; enhances plaque stability	Tao et al., 2020 [174]
pH/ROS dual-responsive cyclodextrin-based nanoparticles (AOCD NP and TAOCD NP)	Rapamycin (RAP)	Targeting vascular inflammatory sites and injured arteries; Type IV collagen-targeted version enhances arterial accumulation	pH and ROS dual-responsive release of rapamycin → inhibits VSMC proliferation and migration; reduces oxidative stress and inflammation; prevents neointimal hyperplasia	Zhang et al., 2020 [175]
Chitosan–fucoidan nanoparticles (CFNs)	No exogenous drug—intrinsic antioxidant and anti-inflammatory activity of fucoidan + chitosan	Targeting P-selectin in atherosclerotic plaques; inhibition of ROS, inflammation, foam cell formation; plaque stabilization	Fucoidan binds P-selectin; CFNs scavenge ROS, reduce IL-6, IL-1β, TNF-α, inhibit foam cell formation, promote plaque stabilization	Liu et al., 2022 [176]
Dextran-mimetic Quantum Dots (Q-Dex)	No exogenous drug—intrinsic macrophage-targeting and imaging capability	Targeting macrophages in inflamed tissues (e.g., visceral adipose tissue, atherosclerotic plaques); multimodal imaging (PET, fluorescence)	Dextran coating enables macrophage-specific uptake via lectin receptors; Q-Dex allows long-circulating, photostable, high-resolution multimodal macrophage imaging	Deng et al., 2022 [177]
Profilin-1 antibody-conjugated, cyclodextrin-modified magnetic iron oxide nanoparticles (RAP@PFN1-CD-MNPs)	Rapamycin	Targeting vascular smooth muscle cells (VSMCs) in atherosclerotic plaques; dual imaging (MRI, NIRF) and therapeutic activity	pH-responsive release of rapamycin in acidic plaque microenvironment → inhibition of VSMC proliferation and migration → increased fibrous cap collagen content → plaque stabilization	Zhang et al., 2020 [178]
CSL112 (human plasma-derived apoA-I + phosphatidylcholine discs)	ApoA-I (native human plasma-derived)	Targets plaque macrophages via ABCA1-mediated cholesterol efflux; promotes plaque stabilization; reduces inflammation	Enhances ABCA1-dependent cholesterol efflux → reduces macrophage lipid content → ↓ inflammation, ↓ foam cell apoptosis, ↑ efferocytosis, ↑ collagen in fibrous cap → stabilizes plaques	Kingwell et al., 2022 [179]

Abbreviations: ABCA1, ATP-binding cassette transporter A1; ABCG1, ATP-binding cassette transporter G1; ACS, acute coronary syndrome; AGEs, advanced glycation end-products; apoA-I, apolipoprotein A-I; CD-MNP, cyclodextrin-modified magnetic nanoparticles; CFNs, chitosan–fucoidan nanoparticles; Col-IV, type IV collagen; CSL112, plasma-derived apolipoprotein A-I plus phosphatidylcholine discs (CSL Behring investigational product); CSNP, cargo-switching nanoparticles; DEX, dexamethasone acetate; DR-NPs, dexamethasone acetate and rapamycin co-loaded mPEG-DSPE calcium phosphate nanoparticles; ECM, extracellular matrix; EES, everolimus-eluting stent; GPx, glutathione peroxidase; HGF, hepatocyte growth factor; ICAM-1, intercellular adhesion molecule 1; IL-1β, interleukin-1 beta; IL-6, interleukin-6; MDA, malondialdehyde; MerTK, MER proto-oncogene tyrosine kinase; MMX, membrane with MXene coating; MMP-2, matrix metalloproteinase-2; MMP-9, matrix metalloproteinase-9; MWCNTs, multi-walled carbon nanotubes; NPs, nanoparticles; pHLIP, pH low-insertion peptide; PNA, peptide nucleic acid; PLGA, poly(lactic-co-glycolic acid); Q-Dex, dextran-mimetic quantum dots; RAP, rapamycin; RCT, reverse cholesterol transport; RES, reticuloendothelial system; rHDL, reconstituted high-density lipoprotein; ROS, reactive oxygen species; S2P, Stabilin-2–targeting peptide; SIRPα, signal regulatory protein alpha; SOD, superoxide dismutase; SWNTs, single-walled carbon nanotubes; TAOCD NP, type IV collagen-targeted pH/ROS dual-responsive cyclodextrin-based nanoparticle; TPI, tyrosine phosphatase inhibitor 1; USPIO, ultrasmall superparamagnetic iron oxide; VSMC, vascular smooth muscle cell.

## Data Availability

All data generated in this research are included within the article.
